# Impact of Maternal Vitamin D Supplementation during Breastfeeding on Infant Serum Vitamin D Levels: A Narrative Review of the Recent Evidence

**DOI:** 10.3390/children9121863

**Published:** 2022-11-30

**Authors:** Shannon DePender, Madeleine M. Russell, Jill DeJager, Sarah S. Comstock

**Affiliations:** Department of Food Science and Human Nutrition, Michigan State University, Lansing, MI 48824, USA

**Keywords:** vitamin D, infants, human milk, breastfeeding, supplementation

## Abstract

Vitamin D supplementation for breastfed infants is recommended due to low levels of vitamin D in human milk and the high prevalence of vitamin D deficiency. The relationship between maternal vitamin D supplementation while breastfeeding and infant serum vitamin D levels is beginning to be described. A literature review was conducted that investigated the impact of maternal supplementation, with at least 4000 IU of vitamin D, on infant serum vitamin D levels. Inclusion criteria were publication between 2016–2022, primary research, exclusively breastfed infants, and mothers taking vitamin D supplements while breastfeeding. Exclusion criteria were publication prior to 2016, review articles, results that did not include infant serum vitamin D levels, and research using participants already included in this review. Over 90% of infants whose mothers took vitamin D supplements while breastfeeding had adequate serum vitamin D levels. The final mean serum vitamin D of all infant participants whose mothers consumed vitamin D supplementation was 66.7 nmol/L, while mean serum vitamin D in those whose mothers did not consume supplements was 33.5 nmol/L. Consumption of vitamin D supplements by lactating women exclusively breastfeeding their infants can lead to adequate serum vitamin D levels in their infants.

## 1. Introduction

Vitamin D supplementation of breastfed infants is recommended by physicians, but this neglects the impact of the breastfeeding parent on infant vitamin D intake. It is the consensus of many organizations worldwide, including the American Academy of Pediatrics, the World Health Organization, and the Dietitians of Canada, that breastfed infants should receive 400 IU of vitamin D daily, due to the high prevalence of vitamin D deficiency (VDD) in breastfed infants secondary to low vitamin D levels in breastmilk [1,2,3]. Human milk is typically low in vitamin D due to limited intake of foods rich in vitamin D and limited sun exposure by the breastfeeding parent [4]. For infants to receive adequate vitamin D while consuming human milk, a vitamin D supplement of 400 IU is recommended by the previously listed organizations to be provided directly to the infant [1,2,3]. This current recommendation does not consider the cause of VDD in infants, which is related to low intake of vitamin D by the lactating parent. Future recommendations to improve breastfed infant vitamin D status should consider recommendations to improve vitamin D levels in the human milk that the infant consumes, perhaps through supplementation of the lactating parent.

VDD is a serious condition, especially in infants; inadequate vitamin D is estimated to occur in 60% of children worldwide [5]. Vitamin D deficiency that remains untreated can impact metabolism and absorption of vitamins and minerals, cause low bone density, and prevent muscles (including the heart) from working efficiently [5]. Infants must receive an adequate source of vitamin D in order to grow and develop appropriately. VDD in infants is defined as serum vitamin D levels less than 37.5 nmol/L, and adequate vitamin D levels are greater than 50 nmol/L [5]. Exclusively human-milk-fed infants are reliant on the milk producer, typically their mother, for all their nutrition. This dependence includes receiving vitamin D and can result in VDD when the lactating individual is deficient.

Adequate vitamin D can be difficult for the breastfeeding parent to achieve. Vitamin D is naturally found in foods such as salmon, beef liver, and egg yolks [6]. Vitamin D may also be found in fortified foods such as orange juice, bread, plant-based milks, and cow’s milk, although this may not be the case in all locations or for all products [6]. Due to the limited amount of vitamin D in everyday foods, lactating people often fail to consume enough vitamin D to make their milk an adequate source of vitamin D for the infant. However, if the lactating individual consumes vitamin D supplements, they may be able to improve vitamin D levels in the milk, making it a better source of vitamin D for the infant. 

A previous review concluded that supplementation of breastfeeding people does not improve vitamin D status of the human-milk-fed infant [7]. That review included papers published between 1982 and 2019. Notably, multiple studies included in that review included daily vitamin D doses of less than 2000 IU [7]. In this paper, we review the relationship between infant serum vitamin D levels and maternal vitamin D supplementation while breastfeeding. This literature review includes articles published between January 2016 and June 2022 that focus on interventions to improve exclusively breastfed infants’ serum vitamin D levels. 

## 2. Materials and Methods

Searches were conducted between 19 May 2022 and 9 June 2022. Primary research articles were identified using PubMed and Web of Science. Search terms were “breastfeeding”, “maternal supplementation”, and “vitamin D”. These terms were searched with the following strings: breastfeeding AND vitamin D supplementation, vitamin D AND maternal supplementation, and maternal vitamin D supplementation while breastfeeding OR breast feeding OR breast milk. Inclusion criteria were publication between January 2016–June 2022, primary research articles, exclusively breastfed infants, and mothers who were taking vitamin D supplements while breastfeeding. Exclusion criteria were publication prior to 2016, review articles, results that did not include infants’ final serum vitamin D levels, and research using participants already included in this review. Study variables included the following: exposure dose of supplementation, sun exposure, and dosing regimen. The outcome variable of interest was infant serum vitamin D levels. After the articles were identified using the defined search terms and databases, the authors reviewed the titles and abstracts to determine which publications met the inclusion or exclusion criteria. Full texts of the reduced set of manuscripts were then reviewed to ensure all inclusion and exclusion criteria were appropriately met (Appendix A).

## 3. Results

A total of 132 research articles were identified using the search methods described above. On review of the abstracts, nine articles fulfilled the criteria for the literature review. After the full text review, three articles were identified that did not meet the criteria for inclusion in the review for various reasons. The six papers used in the final review are summarized in Table 1. 

Within the six articles that fit the inclusion criteria of this literature review, over 90% of infants whose mothers took various doses of vitamin D supplements while breastfeeding had adequate serum vitamin D levels [8,9,10,11,12,13]. The various doses of vitamin D supplements ranged from 4000 IU to 6400 IU daily, with one study providing vitamin D supplements as a bolus of 60,000 IU at four time points [8]. The final mean serum vitamin D concentration of all infant participants whose mothers were receiving vitamin D supplementation was 66.69 nmol/L [8,9,10,11,12,13]. The final mean serum vitamin D concentration of all infants who were not receiving vitamin D supplementation, either directly or indirectly through breast milk, was 33.5 nmol/L [8,12,13]. The studies included in this literature review used a variety of different maternal vitamin D doses and regimens to provide the supplements.

Within the papers presented herein, 912 infants completed their respective studies. Of these infants, 424 received human milk from mothers who took vitamin D supplements [8,9,10,11,12,13]. Infants who were indirectly provided with vitamin D through breast milk had higher serum vitamin D than infants who did not receive vitamin D supplementation either directly or indirectly [8,9,10,11,12,13].

Of the 424 infants who completed the studies, 150 received breast milk from mothers who were taking vitamin D supplementation for longer than 6 months [11,13]. The two studies that researched the effect of maternal vitamin D supplementation on infant serum vitamin D levels for more than 6 months while breastfeeding resulted in final infant serum levels of 91.25 nmol/L and 60.8 nmol/L, respectively [11,13]. The maternal supplementation provided in both studies was 4000 IU daily [11,13]. In the study where the average infant final serum vitamin D level was 60.8 nmol/L, sun exposure was limited to 15 min daily. However, in the study where the average infant final serum vitamin D level was 91.25 nmol/L, the researchers did not take sun exposure into account [11,13]. Two studies investigated vitamin D supplementation for longer than six months, and only one of those two studies took into consideration sun exposure, an important determinant of vitamin D status.

Infants who were breastfed by mothers who took high doses of vitamin D supplements (greater than 6000 IU daily [9,10,11]) had higher serum vitamin D levels than those whose mothers took low-dose vitamin D supplements (Table 2) [8,12,13]. High-dose supplements were delivered either by unsupervised daily supplementation, or by supervised monthly boluses. Infant final serum vitamin D levels were independent of supplementation supervision regimen. Maternal dose of vitamin D supplementation determined final infant vitamin D serum levels. In summary, the higher the levels of supplementation, the higher were the resulting infant serum vitamin D levels [8,9,10,11,12,13]. 

## 4. Discussion

In this review, it was found that infants who were exclusively fed human milk from mothers who took vitamin D supplements had adequate serum vitamin D levels. According to the studies reviewed herein, maternal vitamin D supplementation of at least 4000 IU resulted in adequate levels of vitamin D in their breastfed infants, regardless of the average daily dose or the specific regimen by which the supplementation was administered. There is strong evidence that maternal vitamin D supplementation at the doses and regimens employed in the reviewed manuscripts is an effective method by which to increase infant serum vitamin D levels.

VDD is a common nutrient deficiency in breastfed infants around the world, with the highest rates found in Middle Eastern and south Asian countries. India is estimated to have 90% of infants deficient in vitamin D, while the United States averages 46%, Germany averages 69%, and Australia averages 40% infant vitamin D deficiency [3,5,15]. The majority of identified research studies (four of the six) included in this review were conducted in India, where there is a high prevalence of VDD within the population. Although VDD in breastfed infants is common throughout the world, few interventions have been conducted recently to determine impact of maternal supplementation on infant vitamin D status in most locations worldwide. Thus, given the prevalence of VDD and the lack of recent trials in geographically dispersed locations, it is imperative to evaluate interventions to increase serum vitamin D levels in exclusively breastfed infants in a variety of settings. 

Vitamin D levels in human milk increase with maternal intake [14], therefore the upper limit for vitamin D intake needs to be considered when providing the breastfeeding parent with vitamin D supplementation. The upper limit of intake for vitamin D in infants up to six months of age is 1000 IU per day, and for infants from six months to a year the upper limit of vitamin D is 1500 IU daily [16]. Adults have an upper limit of vitamin D intake of 4000 IU daily, although this amount is debated within the medical community, with amounts up to 10,000 IU daily being noted not to cause toxicity [16]. Maternal vitamin D absorption and the amount transferred through breastmilk to infant varies by individual [17]. Breastfeeding individuals who are not deficient in vitamin D and receive 400–2000 IU daily may only transfer 50–80 IU to their infant [17]. Therefore, there is limited concern that infants could consume toxic levels of vitamin D from human milk. Although maternal vitamin D supplementation does increase the amount of vitamin D infants receive, the amount is sufficiently low that toxicity is unlikely [17].

The articles identified in this review indicate a strong relationship between maternal vitamin D supplementation while breastfeeding and adequate vitamin D levels in the breastfed infant [8,9,10,11,12,13]. Over 90% of exclusively breastfed infants who were receiving breast milk from mothers taking vitamin D supplements were found to have adequate levels of serum vitamin D [8,9,10,11,12,13]. These results suggest that, although it is typically recommended that infants should receive the vitamin D supplement, it could also be considered that breastfeeding mothers take vitamin D to increase maternal and infant vitamin D levels. Maternal supplementation of vitamin D can help increase infant vitamin D levels in those receiving human milk from their breastfeeding parent [8,9,10,11,12,13]. While it is more direct to supplement the infant, some mothers may prefer to avoid supplementation of their infant while being amenable to consuming vitamin D supplements themselves. Thus, supplementation of the breastfeeding parent might prevent VDD in some cases. 

One previously published article reviewed maternal vitamin D supplementation and the effect of such supplementation on infant serum vitamin D levels [7]. That literature review was published in 2020 and featured different inclusion criteria, including a meta-analysis and articles published between 1982–2017 [7]. Their results suggested that there was no overall significant increase in breastfeeding infants’ serum vitamin D levels during maternal vitamin D supplementation, as the weighted mean difference between the control groups and the intervention groups was −1.16 nmol/L [7]. There was an observed increase in infant serum (22.5 nmol/L) relative to controls when mothers were supplemented with over 2000 IU daily over 20 weeks [7]. Neither the dose nor the duration of the intervention had a strong impact on the levels of vitamin D in the serum of the infants [7]. This differs from the literature presented herein, in which a dose relationship was established between maternal vitamin D supplementation and infant serum vitamin D levels. The discrepancies between these two review articles could be attributed to several factors, including the gap of publication years and the doses of vitamin D given to the breastfeeding mothers. 

This literature review has many strengths. Five out of six of these articles were double-blinded studies. Double-blinded studies create less room for bias, as the participant and researchers are both blinded to the dose of vitamin D that each participant receives. Another strength of this review is that four of the studies included a placebo group. This literature review only included studies published within the past six years, to avoid outdated information and research. A limitation of this literature review is that maternal and infant sunlight exposure was not considered across all studies but may play a role in infant serum vitamin D levels [14,18]. Access to sun exposure, or lack thereof, is also dependent on lifestyle factors, such as occupation or style of dress, which were not examined within the context of this review. Each of the studies mentioned sunlight as a source of vitamin D, but few measured or verified sun exposure [8,9,10,11,12,13]. Two studies considered maternal sunlight exposure [11,13], and infant sun exposure was only considered in one of the primary research articles reviewed [12]. Furthermore, there is the potential for limited adherence to taking vitamin D supplements while breastfeeding [19,20]. The interventions in three of the research articles that were included in this literature review provided bolus amounts of vitamin D to the breastfeeding parent, while the other three articles included interventions using daily oral supplementation. Bolus regimens may be recommended due to the possibility of limited compliance to supplementation, although the evidence from this review of the literature indicates that either dosing regimen is effective.

Biases may be present when conducting research. One potential bias observed was the involvement of a single researcher in two of the studies. Another possible bias was the lack of contradictory articles published within the past five years. Results of this literature review show a relationship between maternal vitamin D supplementation while exclusively breastfeeding and adequate vitamin D levels in the infant. Although the evidence suggests that maternal vitamin D supplementation provides adequate vitamin D for the infant, no articles were published within the time frame of this literature review that failed to support the hypothesis that supplementation of the breastfeeding parent could increase serum vitamin D levels in the exclusively human-milk-fed infant. The lack of contradictory articles may be due to possible bias on the part of researchers, as they may fail to publish results that do not support the positive impact of supplementation.

Several factors play a role in serum vitamin D levels in infants. Not only is oral intake vital for adequate vitamin D levels, but sunlight also plays an important role. It is recommended by governmental agencies to receive at least 15 min of direct sunlight daily, with a maximum of 30 min to avoid adverse effects [21]. The amount of sun exposure needed is dependent on a multitude of variables including the amount of melanin in the skin, an individual’s distance from the equator, the time of year, and the amount of skin exposed [21]. Maternal and infant sun exposure as well as maternal vitamin D intake should be considered when determining the need for maternal vitamin D supplementation to increase infant serum vitamin D levels. 

Future research can be conducted to determine the recommended amount of maternal vitamin D supplementation for infants to receive adequate intake through breast milk. As the results from this literature review suggest, it would be beneficial for medical providers and nutrition specialists to educate mothers on the importance of vitamin D supplementation while breastfeeding. Currently, the recommendation is to provide infants with 400 IU of vitamin D daily [1]. Based on the research and the results of this literature review, future recommendations may be to provide vitamin D supplementation to the breastfeeding parent to be transferred through human milk to the infant. Future research is necessary to quantify the dose of supplementation needed for breastfeeding parents to raise vitamin D levels in infant serum. 

## 5. Conclusions

Exclusively breastfed infants can achieve adequate serum vitamin D levels when lactating individuals are supplemented with at least 4000 IU of vitamin D. Supplementation of the lactating individual can potentially improve both infant vitamin D levels and vitamin D levels in the lactating person. Lactating individuals who are reticent to give a vitamin D supplement directly to their infant could be counseled to take a vitamin D supplement at a dose of at least 4000 IU daily to promote adequate infant serum vitamin D levels.

## Figures and Tables

**Table 1:** Summary of the six primary research articles included in this literature review.

Author, Publication Year	Sample Size	Years of Study	Location	Study Type	Intervention	Final Infant Serum Vitamin D Levels (nmol/L) ^1^
Trivedi, 2020 [8]	132 mother/baby pairs recruited; 114 dyads completed the study	2014–2015	India	Randomized double-blind placebo-controlled	Maternal vitamin D supplementation while breastfeeding: 60,000 IU immediately postpartum, and 6, 10, and 14 weeks postpartum. Placebo = inert sugar.	47.3 (+/−12.8)
Dawodu, 2019 [9]	420 mother/baby pairs recruited; 190 pairs enrolled; 102 pairs completed the study	2013–2016	Qatar	Randomized, double-blind, placebo-controlled	Maternal vitamin D supplementation while breastfeeding: 6000 IU daily for 6 months postpartum. All mothers were supplemented. The lower dose of supplementation was considered the placebo.	92.2 (+/−35.5)
Wagner, 2020 [10]	564 mother/baby pairs recruited; 419 pairs enrolled in the study; 262 pairs completed the study	2008–2009	United States	Randomized, double-blind, comparative effectiveness ^3^	Maternal vitamin D supplementation while breastfeeding: 6400 IU per day for 6 months starting at 4–6 weeks postparum. Placebo = Bio-D-Mulsion.	107.1 (+/−35.5)
Ramot, 2022 [11]	220 mother/baby pairs enrolled in the study; 199 dyads completed the study	2014–2017	India	Randomized controlled trial	Maternal vitamin D supplementation of 4000 IU/day for 12 months postpartum. No placebo.	91.1 (IQR: 30.5, 139.8) ^2^
Naik, 2017 [12]	130 mother/baby pairs enrolled in the study; 115 dyads completed the study	2013–2014	India	Randomized, double-blind, placebo-controlled	Maternal vitamin D supplementation while breastfeeding: 60,000 IU per day for 10 days postpartum. Placebo = inert sugar.	72.9 (+/−36.6)
Chandy, 2016 [13]	230 recruited mother/baby pairs; 152 dyads completed the study	2012–2014	India	Double-blind, placebo-controlled	Maternal vitamin D supplementation of 4000 IU/day for 9 months. Placebo = sachet.	60.8 (IQR: 41.3, 80.5) ^2^

^1^ Mean ± SD. ^2^ Median, IQR. ^3^ This is a secondary analysis of data collected during the RCT described in [14].

**Table 2:** Infant serum vitamin D levels after maternal vitamin D supplementation while breastfeeding [8,9,10,11,12,13].

	Maternal Vitamin D Supplementation 4000–6000 IU Daily [8,12,13]	Maternal Vitamin D Supplementation >6000 IU Daily [9,10,11]
Infant Serum Vitamin D nmol/L	Average (SD) 60.36 (±12.84)	Average (SD) 96.85 (±8.89)

## Data Availability

Not applicable.

## Appendix A

Full search syntax used for each database.

Databases Searched:

1. PubMed

Date Range: 2016–2022

Search Terms: “Maternal supplementation and breastfeeding and infant serum Vitamin D” OR “maternal supplementation” and “breastfeeding” and “infant serum vitamin D” OR “breastfeeding” and “maternal supplementation” and “vitamin D” OR “vitamin D” and “maternal supplementation” OR “vitamin D and maternal supplementation” OR T“maternal vitamin D supplementation while breastfeeding”.

2. Web of Science

Date Range: 2016–2022

Search Terms: “Maternal vitamin D” and “breastfeeding” and “infant serum” OR “maternal” and “vitamin D” and “breastfeeding” and “infant” OR “breastfeeding” and “supplement” and “vitamin D” and “maternal” and “infant serum” OR “vitamin D and maternal supplementation” and “breastfeeding” or “breast fed” OR “vitamin D” and “human milk” and “infant” and “serum”.
